# Effects of dietary supplementation with hydroponic wheat seedlings on rumen fermentation, meat quality, amino acid and fatty acid contents, and rumen bacterial diversity in sheep

**DOI:** 10.3389/fmicb.2025.1657777

**Published:** 2025-10-03

**Authors:** Yong Tuo, Jinlong Li, Guzalnur Amat, Zhiqiang Cheng, Liangzhong Hou, Changjiang Zang, Tongjun Guo

**Affiliations:** ^1^Feed Research Institute, Academy of Animal Husbandry Sciences, Xinjiang Uygur Autonomous Region, Urumqi, China; ^2^College of Animal Science, Xinjiang Agricultural University, Urumqi, China; ^3^Xinjiang Key Laboratory of Herbivorous Livestock Feed Biotechnology, Urumqi, China

**Keywords:** hydroponic wheat seedlings, Hu sheep, rumen bacteria, meat quality, fatty acids, economic benefits

## Abstract

Hydroponic wheat seedlings—produced by cultivating wheat seeds in water for seven days—represent a sustainable feed resource for modern livestock farming. Rich in nutrients and bioactive compounds, this innovative fodder exhibits excellent palatability and digestibility, potentially enhancing livestock productivity. This study evaluated the effects of replacing part of the basal diet with hydroponic wheat seedlings (HWS) on ruminal fermentation, bacterial diversity, slaughter performance, and meat quality in finishing Hu sheep. The experiment followed a completely randomized design with fifty healthy 4-month-old Hu ram lambs (27.93 ± 2.16 kg) randomly allocated to five dietary treatments: a control group (CON) fed a basal diet and four experimental groups in which 5%, 10%, 15%, and 20% of the basal diet (dry matter basis) was replaced with HWS (denoted as T5, T10, T15, and T20, respectively). The experiment lasted for 70 days. Days 1–10 were the adaptation period, days 11–70 were the test period. Rumen fluid analysis revealed increased ammonia nitrogen (NH_3_-N) concentrations in HWS groups, particularly at 10%, 15%, and 20% (*P* < 0.01). Dominant bacterial phyla included Bacteroidetes and Firmicutes, with *Prevotella* and *Succiniclasticum* being the most abundant genera. HWS supplementation significantly increased live weight before slaughter and carcass weight (*P* < 0.01), with T15 and T20 exhibiting larger eyes muscle area (*P* < 0.01). Slaughter profits were higher in all HWS groups, peaking in T15. Meat quality analysis showed decreased water loss rate in T10 and T15 (*P* < 0.05), while cooked meat percentage increased across all HWS groups (*P* < 0.01). Inosine monophosphate content decreased in T20 (*P* < 0.05). Heptadecanoic acid (C17:0) content varied among HWS groups (*P* < 0.05). In conclusion, a 15% HWS inclusion optimized ruminal fermentation, microbial composition, and longissimus dorsi quality, affording the highest cost effectiveness for Hu sheep.

## Introduction

Roughage plays a pivotal role in mutton sheep production systems, as high-quality roughage resources provide abundant nutrients to enhance growth performance and ensure healthy development. Roughages typically constitute 20–60% of ruminant diets, and their characteristics and costs are critical determinants of production performance, product quality, and profitability in livestock farming (Yi et al., 2022; Patra, 2012). However, the escalating demand for intensive animal production has exacerbated shortages of premium roughage resources, posing a significant constraint to sustainable livestock industry development. Current livestock production systems employ three primary strategies to secure high-quality roughage: (1) utilization of fermented crop straw (Adesogan et al., 2019), (2) increased integration of whole-plant corn silage (Ferraretto et al., 2018), and (3) incorporation of agricultural byproducts (e.g., distillers' grains) as alternative feed ingredients (Niroula et al., 2019). Nevertheless, these approaches face inherent limitations: fermented straw retains high lignin concentrations, large-scale corn silage production may intensify competition between human and livestock feed resources, and the supply of agricultural byproducts such as distillers' grains often proves inconsistent.

In comparison, hydroponic wheat seedlings (HWS) provide 15.83% crude protein and 4.74% crude fat on a dry matter basis, with only 2.3% lignin content. Its tender and succulent stems and leaves ensure high digestibility, making it an ideal green forage (Sneath and Mcintosh, 2003). This system offers three key advantages: (1) year-round sustainable production, (2) conservation of arable land and water resources, and (3) an environmentally friendly production process with reduced labor requirements (Kkalandarov and Abdullaeva, 2022). While HWS represents an excellent strategy for high-quality roughage provision, its utilization requires careful consideration of production needs and economic returns due to its high moisture content (≥80%) and elevated production costs (dry matter conversion costs significantly exceed those of conventional forages like alfalfa).

During hydroponic cultivation, starch and DM content decline progressively, while protein and lipid synthesis increase, enriching energy substrates for livestock (Chavan and Kadam, 1989). HWS has elevated concentrations of vitamin C, α-tocopherol, γ-tocopherol, β-carotene, and ferulic acid compared to traditional roughage (Yang et al., 2001). Germination of wheat seeds enhances the content of antioxidant compounds, such as vitamins, flavonoids, and phenolic acids in seedlings. By optimizing sprouting duration, the contents of riboflavin, α-tocopherol, and total phenolics can be amplified, thereby strengthening free radical scavenging capacity in herbivores—a critical strategy for maintaining livestock health and productivity (Wang et al., 2024). Studies have demonstrated the efficacy of HWS in enhancing production. During the 30-day feeding trial, supplementation with HWS on a dry matter (DM)-matched basis significantly improved average daily gain in sheep compared to traditional feeding regimens (Gebremedhin et al., 2015). During the 36-day feeding trial, dietary supplementation with 5–15% hydroponic barley seedlings significantly increased total milk protein content, antioxidant activity, and milk fat percentage in lactating ewes. However, at a 20% inclusion level, both milk antioxidant activity and fat content showed reductions (Ma et al., 2024). During the 8-week feeding period, inclusion of 25% HWS in Holstein dairy rations significantly elevated dry matter (DM) intake, average daily gain, milk yield, and milk fat percentage compared to the control group (Bari et al., 2020). Furthermore, an 8-week feeding trial revealed that inclusion of 25% hydroponic barley seedlings in the diet showed no significant effects on feed intake or body weight in Holstein heifers. However, at a 50% inclusion level, significant reductions were observed in both feed intake and body weight, along with decreased apparent digestibility of crude protein and organic matter (Ren et al., 2022). Additional studies revealed that during a 3-week experimental period, replacing 10% of conventional TMR with either hydroponic barley seedlings or HWS improved production efficiency in dairy cows. The hydroponic barley seedlings group achieved the highest feed-to-milk conversion ratio, while the HWS group demonstrated superior body weight gain. Furthermore, the HWS group showed significantly greater apparent digestibility of organic matter, crude protein, and starch compared to both the hydroponic barley seedlings and conventional TMR groups (Zang et al., 2024). Additionally, HWS offers an economic advantage over hydroponic barley seedlings in terms of production cost, as the raw material price of wheat (4.66 CNY/kg) is considerably lower than that of barley (6.72 CNY/kg). Currently, limited information exists regarding the effects of hydroponic wheat seedlings on slaughter performance, rumen fermentation, and microbial community dynamics in finishing Hu sheep. We hypothesize that graded replacement of conventional feed with HWS optimizes rumen function through coordinated restructuring of microbial communities and activation of key metabolic pathways. Therefore, the objective of this study was to evaluate the responses of 4-month-old Hu sheep during the fattening period, while their diets are replaced with different proportions (5–20%) of HWS.

## Materials and methods

The All animal care and handling procedures in this study were conducted in strict accordance with the Guidelines for the Care and Use of Laboratory Animals in China and were approved and overseen by the Animal Care and Use Committee of Xinjiang Academy of Animal Sciences (Approval No. FRI-2023002; Urumqi, China).

### Experimental materials

**Figure 1:**
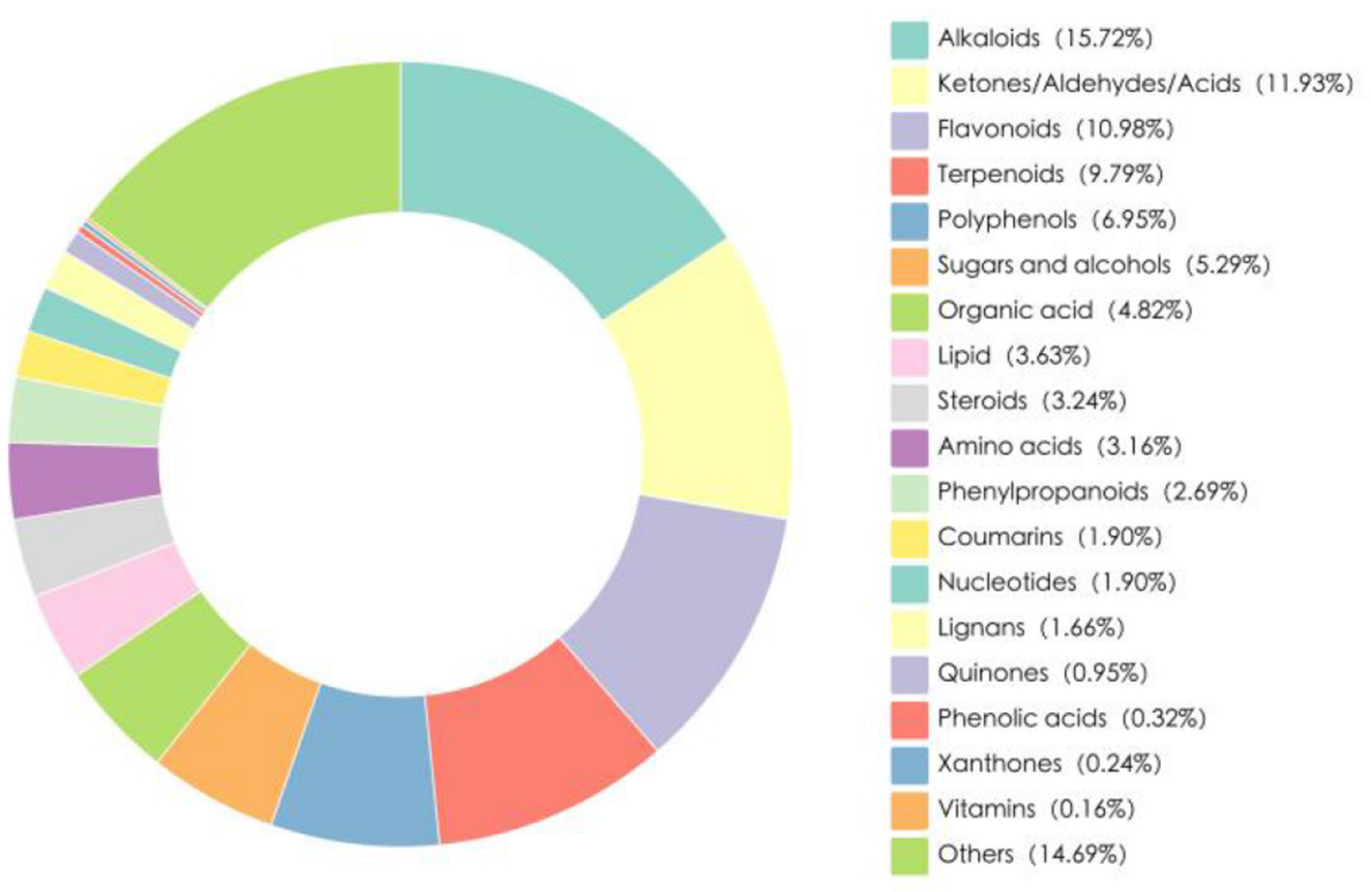
The two-dimensional pie chart of the number of species of active metabolites in HWS.

The hydroponic wheat seedlings (HWS) used in this research were provided by Xinjiang Kemite Agricultural Technology Co., Ltd. (China). They were germinated from red wheat seeds (Triticum aestivum L.) in fully automated containerized cultivation chambers over a 7-day period, Fresh HWS batches were delivered daily to the experimental site, with their nutritional composition shown in Table 1. Bioactive compounds in HWS were analyzed by Biomarker Technologies Corporation (Beijing, China) using liquid chromatography-mass spectrometry (LC-MS), which identified 1266 distinct bioactive substances categorized into 19 classes (Figure 1). Alkaloids constituted the largest proportion (15.72%), followed by keto-aldehydic acids (11.93%), flavonoids (10.98%), terpenoids (9.79%), and polyphenols (6.95%).

**Table 1:** Hydroponic wheat seedlings' conventional nutritional content (dry matter basis%).

**Item**	**Nutritional content**
Dry matter	18.89
Crude protein	15.83
Crude fat	4.74
Neutral detergent fiber	31.12
Acid detergent fiber	18.40
Calcium	0.49
Phosphorus	0.79
Gross energy (MJ/kg)	18.19

Except for dry matter, the content of other nutrients is based on dry matter.

### Animals and experimental design

Fifty genetically homogeneous, healthy 4-month-old Hu rams (initial body weight: 27.93 ± 2.16 kg) were selected from the Shufu County Mutton Sheep Breeding Center (Kashgar, Xinjiang, China) and randomly assigned to five dietary treatment groups (*n* = 10/group) in a completely randomized design (CRD). The nutrient requirements of fattening sheep were formulated according to feeding standards for meat-producing sheep and goats in (NRC 2007). The control group (CON) received a basal total mixed ration (TMR; Table 2), while experimental groups were fed diets in which 5% (T5), 10% (T10), 15% (T15), or 20% (T20) of the basal diet (dry matter basis) was replaced by HWS, with complete nutritional profiles outlined in Table 3. The 70-day trial comprised a 10-day adaptation phase followed by a 60-day experimental period.

**Table 2:** Composition of the basal diet.

**Item**	**Content**
Ingredient, % of DM	
Corn stalks	32.00
Alfalfa hay	10.00
Corn	30.00
Cottonseed meal	19.50
Wheat bran	4.00
NaHCO_3_	0.70
NaCl	0.80
Premix^a^	3.00
Total	100.00

^a^Concentration per kilogram of premix DM: 100 00IU of vitamin A, 40 000IU of vitamin D3, 500 IU of vitamin E, 2 000mg of iron, 1 600 mg of zinc, 800 mg of manganese, 280 mg of copper, 75 mg of selenium, 70 mg of iodine, 40 mg of cobalt.

**Table 3:** Diet nutrient levels.

**Item**	**CON**	**T5**	**T10**	**T15**	**T20**
**Nutrient level, % of DM** ^a^					
Gross Energy (MJ/kg)	16.56	16.64	16.74	16.81	16.88
Crude protein	14.92	14.97	15.02	15.06	15.10
Crude fat	2.27	2.40	2.54	2.65	2.75
Neutral detergent fiber	39.17	38.70	38.17	37.77	37.40
Acid detergent fiber	21.79	21.61	21.42	21.26	21.13
Calcium	0.89	0.87	0.85	0.83	0.81
Phosphorus	0.34	0.36	0.39	0.41	0.43

^a^Nutritional levels were measured values.

All animals were individually housed in pens under standardized environmental conditions. Fresh HWS were transported daily to the experimental facility, mechanically processed using a forage shredder (Model DL-100; Tiejia Agricultural Machinery Manufacturing Co., Ltd., Shandong, China), and thoroughly mixed with other feed ingredients to formulate sheep diets. Feed was administered twice daily (08:00 and 17:30), with daily feed quantities adjusted based on prior intake to maintain residual feed quantities ≥5% of daily provision. Animals had *ad libitum* access to feed and water, with housing maintained under dry and ventilated conditions throughout the trial.

### Production data and sample collection

Throughout the trial, daily feed provision and residual quantities were weighed to calculate feed intake and cost efficiency. All lambs were weighed prior to morning feeding on days 1 and 70. Previous studies have demonstrated a significant correlation between sex and growth performance as well as nutrient metabolism in sheep, with male lambs exhibiting higher growth rates than females during the early developmental stages. To eliminate potential sex-related variations, this study exclusively utilized male lambs (Washaya et al., 2023). On the final trial day, following a 12-h fast and 2-h water restriction period, rumen fluid was collected from five randomly selected sheep in each group. The collection was performed via oral insertion of a rumen sampling device (Anscitech Co., Ltd., Wuhan, Hubei, China) using negative pressure aspiration. To minimize salivary and mucosal contamination, the initial 50 mL aliquot was discarded. The remaining fluid was filtered through four layers of sterile gauze after triplicate pH measurements using a portable pH meter (Anscitech Co., Ltd., Wuhan, Hubei, China). Filtered rumen fluid was aliquotted into four sterile cryovials (10 mL each): one aliquot was preserved in liquid nitrogen for bacterial diversity analysis, and three were stored at −20 °C for subsequent NH_3_-N and volatile fatty acid quantification (Min et al., 2024).

Following rumen fluid collection, lambs were transported to an approved abattoir (Shufu County Xiamushati Breeding Farmers' Professional Cooperative Designated Sheep and Cattle Slaughterhouse. Village 1, Wukushak Town, Shufu County, Kashgar Prefecture, Xinjiang, China) and humanely stunned using an electrical method prior to exsanguination. After exsanguination, carcasses were processed by removal of heads, hooves, hides, and viscera. After carcass weighing and 45-min suspension, the longissimus dorsi muscles were excised from the 12th−13th rib interface, subdivided into standardized sections, flash-frozen in liquid nitrogen, and stored at −80 C for physicochemical quality assessments.

### Indicator measurements

#### Feed samples

During the trial period, HWS and feed samples from each group were collected every 7 days. After the trial concluded, 500 g of each sample was divided into four portions using the quartering method for −20 °C preservation. Moisture content (Method 950.46), ether extract (Method 960.39), crude protein (Method 928.08), crude ash (Method 920.153), total energy (Method 942.05), calcium (Method 968.08), and total phosphorus levels (Method 965.17) were measured according to AOAC methods (Latimer, 2023). Neutral detergent fiber and acid detergent fiber percentages were determined using the methodologies described by (Van Soest et al. 1991).

#### Slaughter performance

Live weight before slaughter (kg) was recorded for all lambs prior to processing. Exsanguination was performed via severance of the carotid arteries and jugular veins, followed by removal of heads, hooves, hides, and viscera. Carcass weight was measured to calculate dressing percentage (carcass weight/live weight before slaughter × 100%). The vertebral column was transected posterior to the 12th rib, and the longissimus dorsi cross-sectional area was traced onto sulfuric acid paper. Loin eye muscle area (cm^2^) was calculated as 0.7 × maximum length × width, measured using digital calipers. GR value (cm), an indicator of carcass fat deposition, was determined as tissue thickness 11 cm lateral to the dorsal midline between the 12th and 13th ribs (Li et al., 2025).

#### Determination of rumen fermentation characteristics

Rumen fluid samples were thawed and centrifuged at 3,000 × g (4 °C, 10 min), and supernatants were collected. A 1 mL aliquot of each supernatant was mixed with 0.25 mL of a standard solution of 25% metaphosphoric acid (w/v) and analyzed for NH_3_-N concentration using a UV-1800 spectrophotometer (Shanghai Mapada Instruments Co., Ltd., Shanghai, China) and the hypochlorite colorimetric method (Mohammed et al., 2004). Volatile fatty acids were quantified using a GC-2010 gas chromatograph (Shimadzu Corporation, Kyoto, Japan) following an established protocol (Broderick and Kang, 1980). Briefly, a 1 mL aliquot of thawed rumen fluid supernatant (obtained by centrifugation at 15,000 × g for 15 min at 4 °C) was transferred to a 2 mL microcentrifuge tube. To precipitate proteins and acidify the sample, 0.2 mL of a metaphosphoric acid solution (25%, w/v) containing an internal standard was added. The internal standard used was 4-methylvaleric acid, at a final concentration of 2 mM. The mixture was vortexed vigorously for 30 s and then incubated at 4 °C for 30 min. Subsequently, the sample was centrifuged at 15,000 × g for 20 min at 4 °C to obtain a clear supernatant. The derivatized supernatant was transferred to a glass GC vial for analysis. Separation and quantification of VFAs were performed using a Shimadzu GC-2010 gas chromatograph equipped with a flame ionization detector (FID) and a high-polarity capillary column (SP-1000, Agilent Technologies, Inc, Beijing, China). The detailed instrumental parameters were as follows: Injector temperature: 250 °C. Detector (FID) temperature: 260 °C. Carrier gas: High-purity Helium (He). Carrier gas flow rate: 1.0 mL/min (constant flow mode). Injection volume: 1.0 μL (in split mode, with a split ratio of 10:1). Oven temperature program: The initial oven temperature was held at 100 °C for 1 min, then increased at a rate of 10 °C/min to 180 °C, and finally raised at 20 °C/min to 220 °C, with a final hold for 2 min.

#### Sequencing and analysis of rumen microbiota

Genomic DNA for subsequent 16S rDNA sequencing was extracted from rumen fluid samples using the TGuide S96 DNA extraction kit (Tiangen Biotech Co., Ltd., Beijing, China). Nucleic acid concentration was quantified spectrophotometrically using a Synergy HTX microplate reader (BioTek Instruments, Hong Kong, China), with integrity verified by 1.8% agarose gel electrophoresis (Beijing ComWin Biotech Co., Ltd., China). The hypervariable V3–V4 regions of bacterial 16S rDNA were amplified via PCR using the universal primers: 338F (5′-ACTCCTACGGGAGGCAGCA-3′) and 806R (5′-GGACTACHVGGGTWTCTAA-3′).

Amplification products were purified with AMPure XP beads, quantified (Qubit fluorometer, Thermo Fisher Scientific), and pooled in equimolar ratios for library construction. DNA libraries passing quality control (Agilent 2100 Bioanalyzer) were sequenced on the Illumina NovaSeq 6000 platform (Illumina Inc., San Diego, CA, USA) using paired-end 250 bp chemistry. Raw sequencing data generated across platforms, including Illumina NovaSeq 6000 outputs, underwent primary processing using Trimmomatic v0.33 for adapter removal and quality filtering (sliding window: 4 bp, average Phred score ≥20). Primer sequences were subsequently trimmed using Cutadapt v1.9.1 with strict mismatch parameters (error rate ≤ 0.1), yielding high-fidelity reads for downstream analysis (Hou et al., 2025).

#### Physical and chemical attributes of the longissimus dorsi muscle

The pH45min value of carcass muscle was measured using a portable pH meter (Shanghai Yidian Scientific Instrument Co., Ltd., Shanghai, China) after calibration with standard buffer solutions. Meat color parameters: (L^*^ = lightness, a^*^ = redness, and b^*^ = yellowness) were determined under light-protected conditions using a portable chroma meter (Hangzhou Caipu Technology Co., Ltd., Zhejiang, China). For water loss measurements, 1 cm thick longissimus dorsi muscle samples were excised perpendicular to the muscle fiber orientation and cored with a 3 cm diameter circular sampler (DL-100; Sumspring Sanquan Zhongshi, Shandong, China). The samples were compressed between six layers of filter paper under 35 kg pressure for 5 min, with water loss percentage calculated from pre- and post-compression weights. Cooked meat percentage was determined by sealing meat specimens (3 cm × 3 cm × 5 cm) in plastic bags, placing them in a water-bath heated at 85 °C for 40 min, cooled to ambient temperature, blotted dry, and calculated using: W1 = pre-cooking weight (g) and W2 = post-cooking weight (g). The core formula is: Cooked meat percentage (%) = (W2/W1) × 100% (Ren et al., 2023).

Proximate composition analysis followed AOAC methods: moisture content was determined after oven-drying at 105 °C to constant weight (Method 950.46); crude protein content was measured via the Kjeldahl method (Method 928.08); crude fat content was analyzed by Soxhlet extraction (Method 960.39); ash content was quantified by calculating residual inorganic matter after 4-h incineration in a muffle furnace at 550 °C (Method 920.153) (Latimer, 2023).

#### Amino acid and fatty acid content of the longissimus dorsi muscle

A 100 mg aliquot of freeze-dried longissimus dorsi tissue was thawed at 4 °C, combined with 1 mL of sterile physiological saline solution (0.9% w/v NaCl), and homogenized to complete disruption using a mortar and pestle under liquid nitrogen-cooled conditions. The homogenates were transferred to centrifuge tubes, mixed with 5% sulfosalicylic acid solution, incubated for 10 min, and centrifuged at 13,500 × g (4 °C, 15 min). The supernatants were adjusted to neutral pH with 10% NaOH, filtered through a 0.22 μm membrane, and analyzed for amino acid composition and concentration using an L-8900 high-speed amino acid analyzer (Hitachi High-Technologies Co., Tokyo, Japan). For fatty acid profiling, 50 g samples of freeze-dried longissimus dorsi tissue were thawed at 4 °C, pulverized using a water-cooled knife mill (Foss KN 195 Knifetec, Foss Analytical A/S, Hilleroed, Denmark), and processed according to (Liang et al. 2023). Total lipids were extracted, dissolved in chloroform, and subjected to methyl esterification. Fatty acid methyl esters were analyzed by gas chromatography (Agilent 7890B, Agilent Technologies, USA) and compared with C4~C24 fatty acid methyl ester reference standards (Sigma-Aldrich, Inc., USA), with identification and quantification based on retention time.

### Statistical analysis

Experimental data were processed using Excel 2019 and subjected to statistical analysis with SPSS 23.0 (IBM, New York, USA). All datasets were initially evaluated for normality using the Shapiro-Wilk test. Subsequently, one-way analysis of variance (ANOVA) was performed, followed by multiple comparisons using Duncan's multiple range test. Multiple comparisons of means were performed using Duncan's new multiple range test, a method commonly employed in animal science research for its high power in detecting differences among treatment groups. Results are presented as means ± SEM. Statistical significance thresholds were set at *P* < 0.05 and *P* < 0.01. For dose-response relationships, we performed one-way ANOVA with orthogonal polynomial contrasts to evaluate linear and quadratic trends. Alpha diversity metrics were visualized using GraphPad Prism 8.0 (GraphPad Software, San Diego, USA), while correlation analyses were conducted and graphically represented using Origin 22.0 (OriginLab, Northampton, USA).

## Results

### Effects of hydroponic wheat seedlings on ruminal fermentation parameters in sheep

As shown in Table 4, ruminal NH_3_-N concentration exhibited a linear increase (*P* < 0.01) with escalating HWS inclusion levels. The T15 group demonstrated a significant elevation (*P* < 0.01) in NH_3_-N concentration compared to (CON), while T10 and T20 groups showed significant increases (*P* < 0.05). Notably, T15 and T20 exceeded T5 in NH_3_-N concentration (*P* < 0.05). Both ruminal NH_3_-N and acetate concentrations displayed linear upward trends (*P* < 0.05) with incremental HWS supplementation.

**Table 4:** Effect of substitution level of HWS on rumen fermentation parameters in sheep.

**Items**	**CON**	**T5**	**T10**	**T15**	**T20**	**SEM**	***P*** **-value**		
							**Trt**	**L**	**Q**
pH	6.172	6.232	6.256	6.302	6.246	0.020	0.372	0.135	0.219
NH_3_-N (mg/dL)	18.974^Bc^	19.274^Bbc^	20.342^ABab^	21.140^Aa^	20.620^ABa^	0.243	0.009	0.001	0.250
Acetate (mmol/L)	66.109	67.208	69.725	68.626	68.812	0.444	0.063	0.023	0.113
Propionate (mmol/L)	17.818	18.702	19.155	18.876	18.357	0.384	0.862	0.669	0.313
Isobutyrate (mmol/L)	1.199	1.090	1.022	1.276	1.024	0.040	0.162	0.535	0.907
Butyrate (mmol/L)	13.898	13.209	13.020	14.481	13.404	0.228	0.258	0.856	0.640
Isovalerate (mmol/L)	1.744	1.820	1.616	2.099	1.632	0.070	0.175	0.906	0.479
Valerate (mmol/L)	0.847	0.784	0.740	0.881	0.623	0.036	0.178	0.162	0.479
TVFA (mmol/L)	101.614	102.813	105.278	106.240	103.853	0.602	0.094	0.054	0.072
Acetate/Propionate	3.768	3.669	3.657	3.667	3.753	0.081	0.990	0.959	0.600

^A, B^Different superscripts indicate significant differences within a row (*P* < 0.01).

^a, b, c^Different superscripts indicate significant differences within a row (*P* < 0.05). SEM is the pooled standard error between five groups; the *P*-value indicates significance. Trt, treatment effect; L, linear; Q, quadratic; TVFA, total volatile fatty acids.

### Effects of dietary supplementation with HWS on microbial diversity in the rumen of sheep

#### Venn diagram showing overlap of rumen bacterial taxa across treatments

**Figure 2:**
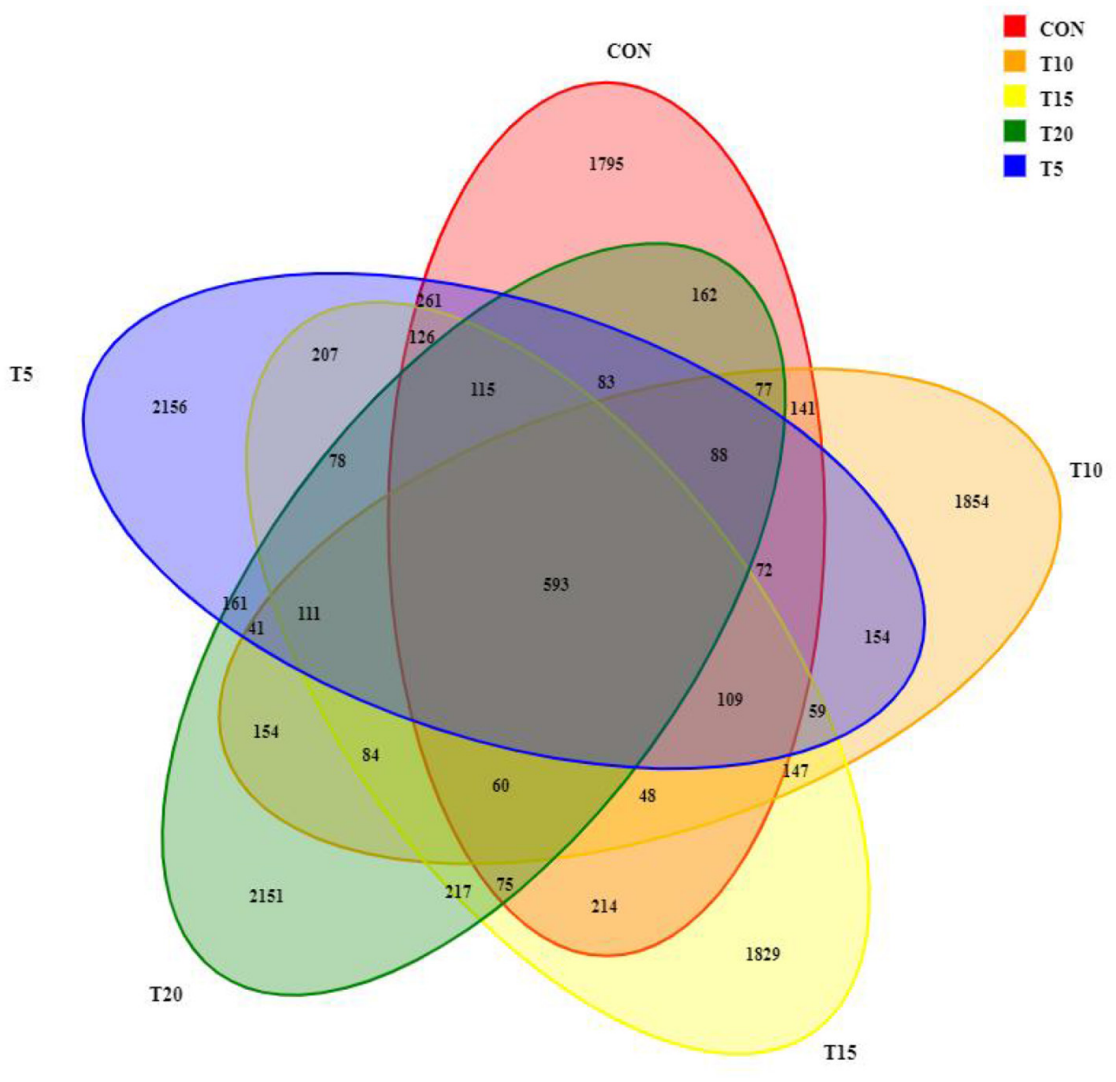
Venn diagram of rumen bacteria.

Rumen fluid sequencing revealed >99% coverage across all samples. A total of 1,970,786 paired-end reads were obtained from 25 samples, yielding 1,962,740 clean reads after quality filtering, with each sample generating ≥50,537 clean reads (mean: 78,510 reads/sample). Sequences were clustered at 97% similarity, identifying 13,422 operational taxonomic units (OTUs) (Figure 2). Among these, 593 OTUs were shared across all groups, while CON, T5, T10, T15, and T20 harbored 1,795, 2,156, 1,854, 1,829, and 2,151 unique OTUs, respectively, with CON exhibiting the lowest OTU.

#### Bacterial composition of rumen fluid

**Figure 3:**
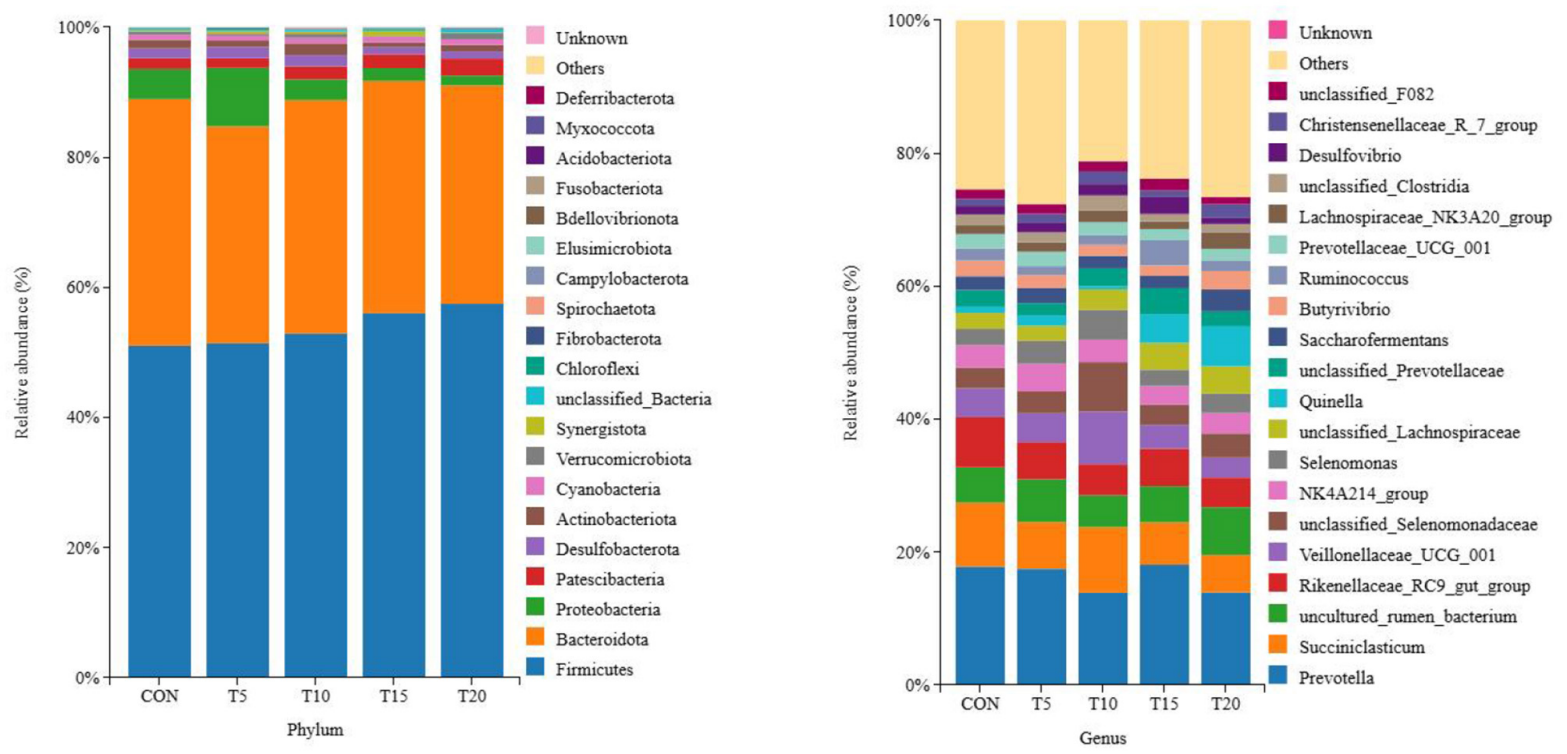
Columnar distribution of species. Distribution of bacterial taxa averaged under phyla and genera levels across the dierent treatment groups. The Bayesian classifier annotates the feature sequences for classification, as a percentage of the total sequence.

As shown in Figure 3, a total of 20 bacterial phyla were identified across the 25 samples, with Bacteroidota, Firmicutes, Proteobacteria, and Desulfobacterota as the four dominant phyla, accounting for 55.06%, 34.45%, 3.36%, 1.83%, and 1.80% of the total sequences, respectively. At the genus level, 416 genera were detected across all samples. The top five genera by relative abundance were Prevotella, Succinivibrio, unclassified genera, Rikenellaceae_RC9_gut_group, and Veillonella_UCG-001, constituting 16.25%, 7.70%, 5.83%, 5.59%, and 4.64% of the total sequences, respectively.

#### Alpha diversity analysis of rumen bacteria

**Figure 4:**
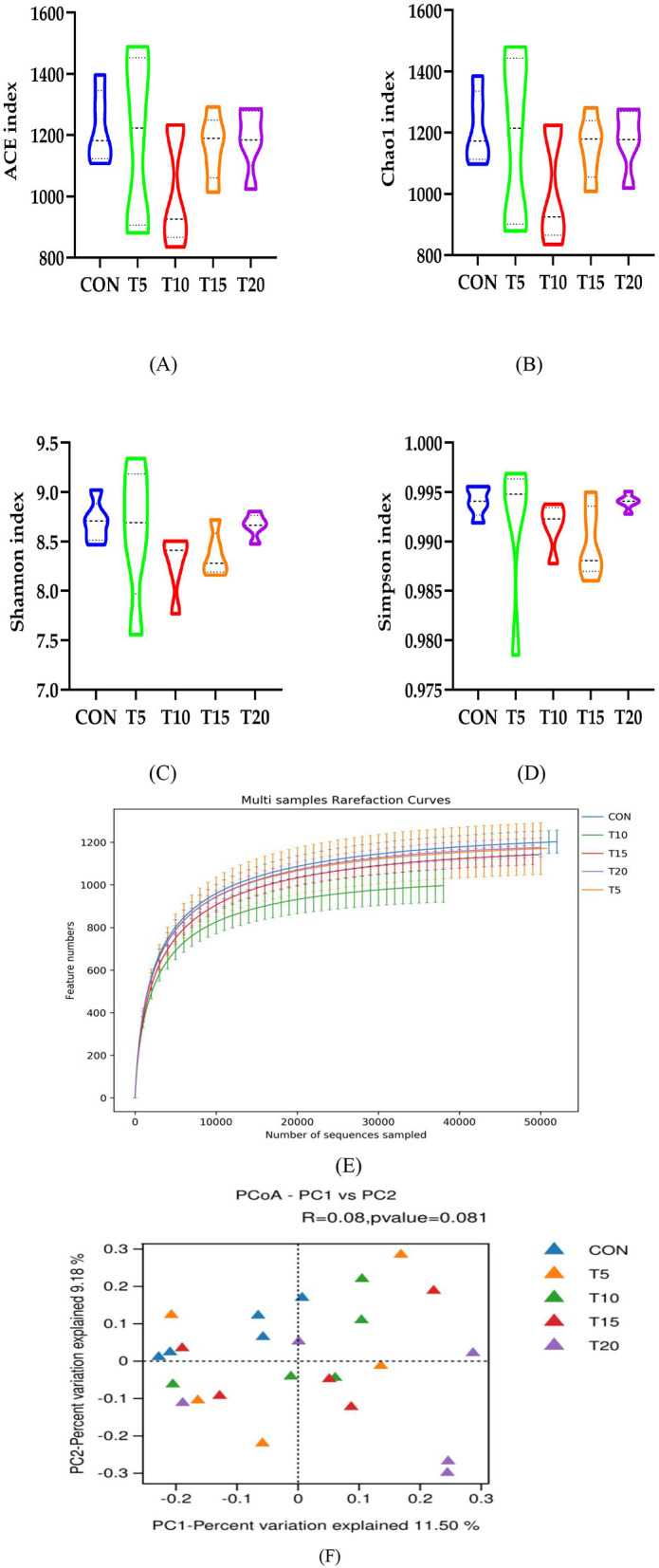
Diversity, abundance, dilution curves and PCoA plots. Figure 4 compares the control group (CON) with treatment groups (T5, T10, T15, T20) through multiple datasets. **(A–D)** Illustrate the variation trends of four different indices (ACE index, Chao1 index, Shannon index, and Simpson index) across the groups. **(E, F)** Depict the distribution of feature numbers for all groups, with both panels sharing the same axis labels and value ranges.

As illustrated in Figures 4A–D, no significant differences (*P* > 0.05) were observed in bacterial richness or diversity between CON and HWS-supplemented groups, with all cohorts maintaining >99% coverage of ruminal bacterial communities. Figure 4E demonstrates robust sequencing depth and accurate taxonomic representation through rarefaction curves, confirming the comprehensive microbial community characterization across samples. Principal coordinates analysis (PCoA) based on Bray-Curtis dissimilarity metrics (Figure 4F) revealed no statistically significant divergence (*R* = 0.080, *P* = 0.081) in ruminal microbiota composition between CON and HWS-supplemented groups.

### Effect of dietary HWS on the slaughter performance and economic benefits from the experimental sheep

As presented in Table 5, the effects of HWS supplementation on ovine slaughter performance were evaluated. Increasing HWS levels induced quadratic enhancements in live weight before slaughter and carcass weight (*P* < 0.05), while linear improvements in dressing percentage and *longissimus dorsi* muscle eye muscle area (*P* < 0.01) were observed. The HWS groups exhibited significantly higher live weight before slaughter than the CON group (*P* < 0.05 or *P* < 0.01), with the T15 group surpassing T10 (*P* < 0.05) and T5 (*P* < 0.01), and T20 exceeding T5 (*P* < 0.01). Carcass weights in the HWS groups were significantly increased compared to CON (*P* < 0.05 or *P* < 0.01), with T15 outperforming T10 (*P* < 0.05) and T5 (*P* < 0.01), and T10 exceeding T5 (*P* < 0.05). Dressing percentages in T10, T15, and T20 groups were significantly higher than CON (*P* < 0.05). The *longissimus dorsi* muscle eye muscle area of T15 surpassed T20 (*P* < 0.05), T10 (*P* < 0.01), T5 (*P* < 0.01), and CON (*P* < 0.01), while T20 exceeded CON (*P* < 0.05). No significant intergroup differences were detected in GR values (*P* > 0.05).

**Table 5:** Effect of feeding HWS on slaughter performance of sheep.

**Items**	**CON**	**T5**	**T10**	**T15**	**T20**	**SEM**	***P*** **-value**		
							**Trt**	**L**	**Q**
LWBS (kg)	35.180^Cd^	36.777^BCc^	37.413^ABbc^	38.980^Aa^	38.273^ABab^	0.380	< 0.001	< 0.001	0.032
Carcass weights (kg)	16.967^Cd^	18.160^BCc^	19.057^ABb^	20.123^Aa^	19.347^ABab^	0.308	< 0.001	< 0.001	0.004
Slaughter rate (%)	48.230^b^	49.373^ab^	50.930^a^	51.637^a^	50.563^a^	0.416	0.041	0.010	0.070
Eye muscle area (cm^2^)	25.717^Bc^	26.133^Bbc^	26.737^Bbc^	28.600^Aa^	27.313^ABb^	0.305	0.002	0.001	0.158
GR value (mm)	21.157	21.220	21.167	21.567	21.117	0.448	0.999	0.944	0.899

^A, B, C^Different superscripts indicate significant differences within a row (*P* < 0.01).

^a, b, c^Different superscripts indicate significant differences within a row (*P* < 0.05). SEM is the pooled standard error between five groups; the *P-value* indicates significance. Trt, treatment effect; L, linear; Q, quadratic; LWBS, live weight before slaughter.

HWS' nutritional favorability and cost-effectiveness as a partial substitute in the basal diet reduced feed cost per kg and enhanced utilization efficiency, thereby lowering production expenses. Table 6 illustrates the economic impact of dietary HWS substitution ion meat sheep production. Based on market prices in Kashgar, Xinjiang, China, replacing 5–20% of basal feed with HWS significantly decreased feed cost per kilogram. The net profits from all HWS groups exceeded those of the CON, but T15 achieved the highest profitability.

**Table 6:** Economic benefit analysis of HWS feeding fattening sheep.

**Items**	**CON**	**T5**	**T10**	**T15**	**T20**
Hot carcass weight (kg/piece)^a^	16.967	18.160	19.057	20.123	19.347
Price of mutton (CNY/kg)^b^	50.000	50.000	50.000	50.000	50.000
Income (CNY/piece)	848.350	908.000	952.850	1,006.150	967.350
Feed unit price (CNY/kg)^c^	2.291	1.992	1.774	1.609	1.482
Total feed intake (kg/piece) (kg)	72.977	107.070	117.563	149.186	163.627
Lamb costs (CNY/piece)^d^	600.000	600.000	600.000	600.000	600.000
Total cost (CNY/piece)	767.190	813.283	808.557	840.040	842.495
Net profit (CNY/piece)	81.160	94.717	144.293	166.110	124.855

CNY, China Yuan.

^a^The carcass refers to the edible portion of a sheep remaining after the removal of the head, hooves, hide, and internal organs during the slaughtering process. The separated internal organs and other by-products are typically purchased by the slaughterhouse to offset the costs associated with the execution of slaughtering procedures and other related operations.

^b^Unit price of mutton is calculated according to the current market price in Kashgar, Xinjiang: 50 CNY/kg.

^c^The price of feed raw materials is calculated according to the current market price in Kashgar, Xinjiang, which is used in the present study as follows: hydroponic wheat seedlings 0.5 CNY/kg, corn straw 0.85 CNY/kg, alfalfa 1.8 CNY/kg, corn 2.65 CNY/kg, cottonseed meal 3.75 CNY/kg, wheat bran 2.8 CNY/kg, calcium carbonate 2.5 CNY/kg, sodium bicarbonate 2.8 CNY/kg, salt 0.85 CNY/kg, premix 5.8 CNY/kg.

^d^The cost of lambs refers to the expense incurred for purchasing weaned lambs prior to the implementation of the experiment. Based on the local market price in Kashgar, Xinjiang, the cost for fattening lambs weighing 25–30 kg was 600 CNY per head.

### Effect of HWS on amino acid and fatty acid composition of *longissimus dorsi* muscle

As presented in Table 7, the effects of HWS substitution on *longissimus dorsi* muscle quality attributes, nutrient composition, and flavor-related compounds in Hu sheep were quantified. Water loss rate and inosine monophosphate content exhibited quadratic decreases (*P* < 0.05), while Cooked meat percentage demonstrated a quadratic increase (*P* < 0.01) with increasing HWS inclusion. The T10 and T15 groups showed significantly reduced water loss compared to the CON (*P* < 0.05). Cooked meat percentage was markedly elevated across all HWS groups vs. CON (*P* < 0.01), with T15 exceeding T5, T10, and T20 (*P* < 0.01), and T10/T20 surpassing T5 (*P* < 0.01). Inosine monophosphate content in T20 was significantly lower than CON and T15 (*P* < 0.05), while T5, T10, and T20 had reduced inosine monophosphate levels relative to T15 (*P* < 0.05). No significant differences were observed for other measured parameters (*P* > 0.05).

**Table 7:** Effect of feeding HWS on the muscular characteristics of the longest back of sheep.

**Items**	**CON**	**T5**	**T10**	**T15**	**T20**	**SEM**	***P*** **-value**		
							**Trt**	**L**	**Q**
pH_45min_	6.117	5.990	6.203	6.117	5.937	0.075	0.848	0.700	0.572
Water loss rate (%)	29.400^a^	29.047^a^	27.103^b^	26.543^b^	28.163^ab^	0.366	0.025	0.022	0.034
Cooked meat percentage (%)	57.660^Dd^	59.593^Cc^	63.977^Bb^	66.790^Aa^	62.673^Bb^	0.875	< 0.001	< 0.001	< 0.001
Luminosity (L^*^)	30.453	28.820	30.283	35.063	29.173	1.338	0.654	0.721	0.670
Redness (a^*^)	16.917	19.193	18.313	15.930	19.007	0.949	0.832	0.904	0.991
Yellowness (b^*^)	19.590	21.437	21.273	23.017	23.923	0.836	0.582	0.126	0.997
Moisture (%)	76.193	75.917	75.277	74.687	75.740	0.302	0.607	0.361	0.329
Protein (%)	20.477	20.197	20.547	20.643	20.467	0.142	0.926	0.717	0.973
Fat (%)	5.187	5.063	5.247	5.013	5.230	0.072	0.846	0.950	0.702
Ash (%)	1.083	1.103	1.097	1.060	1.127	0.011	0.405	0.572	0.487
Thiamine (mg/kg)	1.393	1.240	1.504	1.360	1.373	0.040	0.394	0.781	0.824
Cholesterol (mg/kg)	516.053	502.377	528.890	506.270	522.143	5.402	0.569	0.693	0.836
IMP (mg/kg)	42.955^ab^	33.831^bc^	33.591^bc^	43.881^a^	31.752^c^	1.790	0.047	0.228	0.700

^A, B, C, D^Different superscripts indicate significant differences within a row (*P* < 0.01).

^a, b, c^Different superscripts indicate significant differences within a row (*P* < 0.05). SEM is the pooled standard error between five groups; the *P*-value indicates significance.

IMP, inosine monophosphate; Trt, treatment effect; L, linear; Q, quadratic.

### Effect of HWS on amino acid and fatty acid composition of *longissimus dorsi* muscle

Table 8 delineates the effects of HWS on amino acid profiles in ovine *longissimus dorsi* muscle. Ala, Gln, and His were the predominant contributors to the total amino acid composition. Increasing HWS inclusion resulted in numerical increases in Gly, Ser, Thr, Ile, Asp, Met, and Tyr across all treatment groups (*P* > 0.05). The T5 and T15 groups exhibited higher essential amino acids (EAAs), non-essential amino acids (NEAAs), dibasic amino acids like lysine, arginine and histidine (DAAs), and total amino acids (TAAs) compared with the CON, but no intergroup differences reached statistical significance (*P* > 0.05).

**Table 8:** Effect of feeding HWS on amino acid composition of *longissimus dorsi* muscle in sheep (μg/g).

**Items**	**CON**	**T5**	**T10**	**T15**	**T20**	**SEM**	***P*** **-value**		
							**Trt**	**L**	**Q**
Gly	125.162	162.931	128.446	148.168	129.462	8.373	0.621	0.923	0.443
Ala	508.858	509.381	458.219	549.808	463.598	19.544	0.623	0.737	0.862
GABA	3.751	4.290	4.403	4.159	3.119	0.312	0.753	0.573	0.243
Ser	111.111	149.223	124.545	144.581	120.239	8.635	0.646	0.837	0.318
Pro	52.787	67.867	56.378	59.417	48.404	3.746	0.607	0.548	0.277
Val	76.151	85.020	75.842	78.562	80.556	3.453	0.942	0.934	0.956
Thr	51.268	64.699	55.011	59.768	51.737	3.476	0.767	0.884	0.389
Ile	63.863	74.713	66.878	71.604	64.299	2.768	0.735	0.918	0.365
Leu	144.665	167.969	154.103	160.092	144.309	5.462	0.661	0.838	0.256
Asn	80.376	82.849	74.584	86.124	75.640	2.736	0.706	0.771	0.808
Orn	20.665	29.299	19.130	24.942	19.929	3.376	0.902	0.833	0.730
Asp	18.675	25.030	22.285	27.151	29.944	3.508	0.909	0.400	0.989
Gln	388.091	472.663	387.515	432.237	324.379	24.271	0.422	0.347	0.234
Lys	107.989	113.132	110.565	108.815	97.032	4.172	0.833	0.440	0.412
Glu	158.463	192.915	158.300	153.020	115.050	11.289	0.333	0.129	0.231
Met	59.817	72.321	68.807	69.756	63.866	2.734	0.675	0.793	0.213
His	389.304	413.056	336.367	496.911	368.630	25.980	0.396	0.818	0.761
Phe	88.694	90.645	91.512	92.942	86.128	2.611	0.956	0.896	0.514
Arg	132.707	136.294	139.345	154.983	125.461	5.111	0.507	0.911	0.246
Tyr	78.727	99.575	88.620	93.444	85.018	3.433	0.406	0.793	0.162
Trp	19.466	20.310	18.852	20.452	18.812	0.552	0.857	0.794	0.718
EAA	1,001.217	1,101.863	977.938	1,158.901	975.370	41.936	0.599	0.987	0.488
NEAA	1,285.977	1,450.280	1,245.900	1,401.202	1,142.034	55.016	0.449	0.406	0.314
SEAA	336.596	398.800	356.411	396.596	339.942	14.549	0.543	0.967	0.243
DAA	978.578	1,080.477	947.382	1,064.534	909.200	34.523	0.501	0.546	0.389
TAA	2,680.590	3,034.180	2,639.707	3,036.937	2,515.614	104.515	0.418	0.664	0.294

SEM is the pooled standard error between five groups; the *P*-value indicates significance.

Trt, treatment effect; L, linear; Q, quadratic.GABA, Gamma-aminobutyric acid; EAA, essential amino acids; NEAA, non-essential amino acid; SEAA, semi-essential amino acid; DAA, delicious amino acids; TAA, Total animo acid.

Table 9 details HWS-mediated modifications in fatty acid composition of *longissimus dorsi* muscle. Increasing HWS levels were associated with quadratic elevation in PUFA content (*P* < 0.05), while T5 and T10 displayed significantly higher heptadecanoic acid (C17:0) concentrations than T15 and T20 (*P* < 0.05). Other fatty acid constituents showed no significant intergroup variations (*P* > 0.05).

**Table 9:** Effect of feeding HWS on fatty acid composition of *longissimus dorsi* muscle in sheep.

**Items**	**CON**	**T5**	**T10**	**T15**	**T20**	**SEM**	***P*** **-value**		
							**Trt**	**L**	**Q**
C10:0 %	0.304	0.290	0.227	0.343	0.241	0.025	0.661	0.705	0.993
C12:0 %	0.436	0.403	0.195	0.528	0.244	0.074	0.661	0.651	0.955
C14:0 %	4.855	4.739	3.388	5.350	3.567	0.498	0.730	0.615	0.996
C14:1 %	0.138	0.134	0.092	0.151	0.087	0.017	0.723	0.514	0.892
C15:0 %	0.545	0.498	0.498	0.561	0.444	0.028	0.768	0.533	0.765
C16:0 %	26.504	26.893	26.870	26.489	26.441	0.165	0.879	0.694	0.445
C16:1 %	1.675	1.665	1.642	1.549	1.314	0.065	0.410	0.096	0.358
C17:0 %	1.354^ab^	1.487^a^	1.543^a^	1.279^b^	1.330^b^	0.035	0.049	0.203	0.055
C18:0 %	20.741	20.829	21.236	21.050	23.345	0.493	0.476	0.153	0.380
C18:1n9c %	38.210	36.859	38.053	37.303	37.664	0.341	0.777	0.811	0.645
C18:2n6c %	3.959	4.915	5.399	3.860	4.383	0.274	0.373	0.914	0.225
C20:0 %	0.122	0.113	0.110	0.129	0.115	0.004	0.692	0.957	0.767
C20:1 %	0.064	0.067	0.076	0.065	0.067	0.002	0.557	0.913	0.296
C18:3n3 %	0.909	0.900	0.453	1.145	0.574	0.160	0.717	0.733	0.992
C20:4n6 %	0.184	0.206	0.216	0.201	0.186	0.009	0.855	0.980	0.294
SFA %	54.861	55.254	54.068	55.727	55.725	0.331	0.532	0.380	0.486
UFA %	45.139	44.746	45.932	44.273	44.275	0.331	0.532	0.380	0.486
UFA/SFA	0.824	0.810	0.850	0.795	0.795	0.011	0.520	0.369	0.490
MUFA %	40.087	38.725	39.864	39.067	39.132	0.322	0.700	0.535	0.757
PUFA %	5.052	6.021	6.068	5.206	5.143	0.171	0.118	0.549	0.034

^a, b^Different superscripts indicate significant differences within a row (*P* < 0.05). SEM is the pooled standard error between five groups; the *P*-value indicates significance.

Trt, treatment effect; L, linear; Q, quadratic. SFA, saturated fatty acid; UFA, unsaturated fatty acid; MUFA, monounsaturated fatty acid; PUFA, polyunsaturated fatty acid.

### Relationship between meat quality, ruminal fermentation levels and bacteria

**Figure 5:**
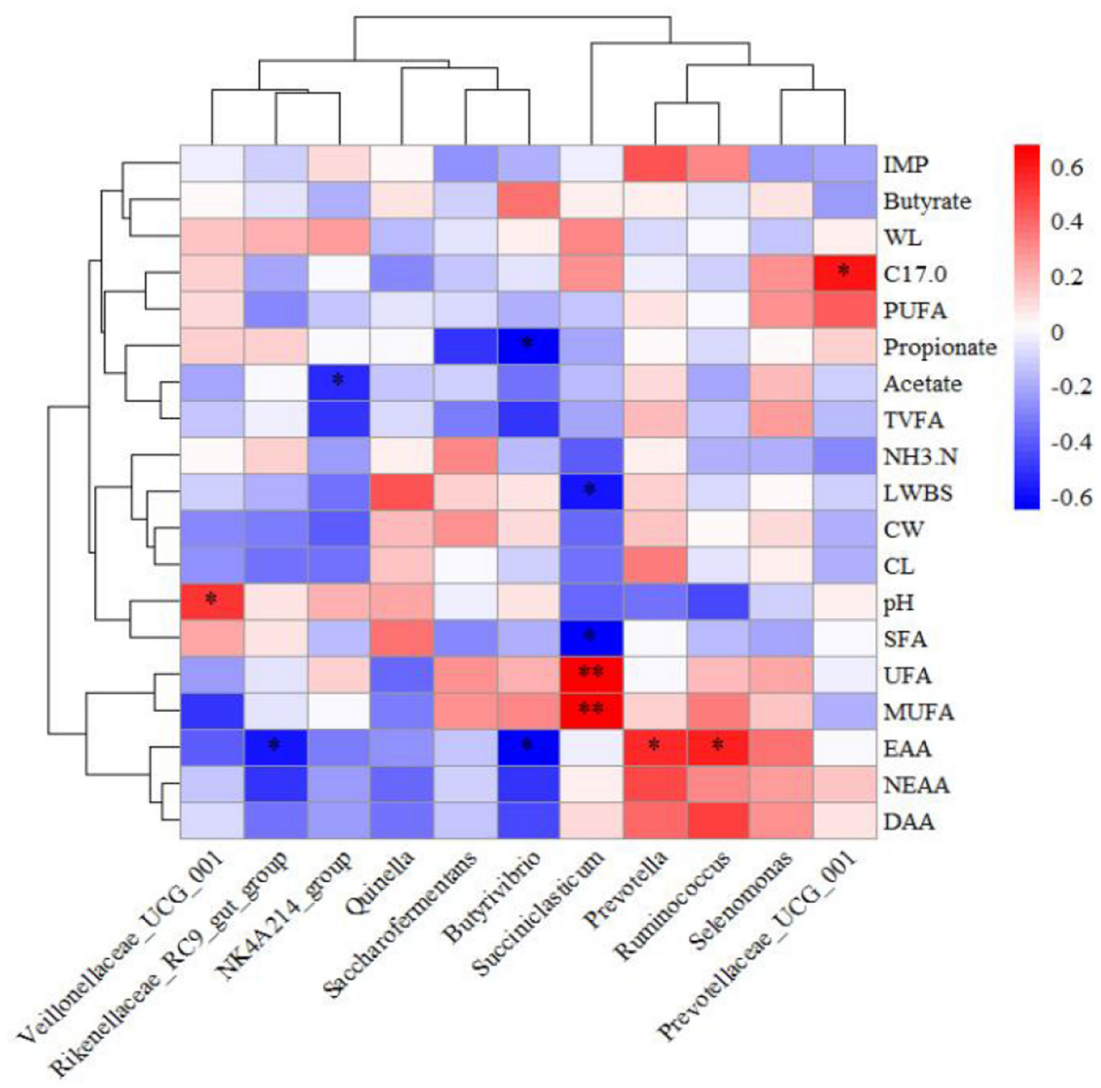
Heatmap showing the relationships among relative microbial abundance, rumen fermentation parameters, and meat quality. IMP, Inosine monophosphate; WL, Water loss rate; C17.0, C17:0; NH3.N, NH_3_-N; LWBS, Live weight before slaughter; CW, Carcass weight; CL, Cooked meat percentage.^*^P < 0.05 ^**^P < 0.01.

Correlation coefficients were calculated using Pearson's method to construct a heatmap elucidating the relationships among ruminal bacteria, fermentation parameters, and meat quality attributes (Figure 5). The ruminal pH exhibited a positive correlation with *Veillonellaceae*_*UCG*_001 (*P* < 0.05). Acetate concentration in the rumen demonstrated a negative correlation with the *NK*4*A*214_*group* (*P* < 0.05), while propionate concentration was inversely correlated with *Butyrivibrio* (*P* < 0.05). C17:0 content in meat showed a positive association with *Prevotellaceae*_*UCG*_001 (*P* < 0.05). EAA content in meat was negatively correlated with the *Rikenellaceae*_*RC*9_*gut*_*group* and *Butyrivibrio* (*P* < 0.05) but positively correlated with *Prevotella* and *Ruminococcus* (*P* < 0.05). *Saccharofermentans* displayed negative correlations with live weight before slaughter and SFA content (*P* < 0.05), while showing positive correlations with UFA and MUFA (*P* < 0.01).

## Discussion

NH_3_-N serves as a critical nitrogen source for microbial protein synthesis, directly influencing the ruminal microbial protein utilization efficiency and microbial proliferation dynamics. While ruminal NH_3_-N levels typically are positively correlated with dietary protein content and intake, this study revealed significant NH_3_-N elevation in the T10, T15, and T20 groups, potentially attributable to HWS's superior protein composition compared to conventional diets (Zhou et al., 2018). The enhanced degradability of hydroponic plant cell walls likely stimulated the fibrolytic activity of rumen bacteria such as *Ruminococcus spp*., accelerating amino acid deamination and microbial protein synthesis efficiency, thereby modulating ruminal ammonia utilization. Dietary inclusion of HWS increased the fiber content, prolonging rumination duration and enhancing feed-specific microbial adhesion, which synergistically improved microbial protein utilization and NH_3_-N production (Min et al., 2024). Notably, although the NH3-N concentration in T20 was higher than in CON, its incremental gain relative to T15 suggests a potential threshold inhibitory effect. This phenomenon may be attributed to excessive alkaloids supplied by the 20% HWS, which could suppress the growth of *Butyrivibrio* in the rumen, thereby reducing amino acid deamination and subsequent ammonia nitrogen production (Petric et al., 2021). Acetate concentration increased linearly with the percentage of HWS inclusion, likely driven by the higher soluble fiber content in HWS, which preferentially favors fibrolytic bacteria such as *Ruminococcus spp*. that generate acetate via enhanced carbohydrate fermentation (Jeyanathan et al., 2014). Concurrently, the trend toward a decrease in acetate-to-propionate ratio suggests metabolic pathway shifts, with propionate's relative predominance potentially enhancing hepatic gluconeogenesis—a physiologically advantageous adaptation for ovine energy metabolism.

The ruminal microbial community in ruminants exhibits a complex and diverse structure including bacteria, fungi, and archaea. Bacteria constitute the most functionally significant microbial group in the rumen, with diversity and relative abundance are strongly influenced by diet (Wang et al., 2017). Rumen homeostasis is contingent upon microbial richness and diversity, with high bacterial richness and diversity being recognized as beneficial for enhancing ruminal stability (Weimer, 2015). The Chao1 and ACE indices reflect microbial richness, while the Simpson and Shannon indices characterize community diversity. In this study, no significant differences were observed in ACE, Chao1, Simpson, or Shannon indices across dietary treatments. These findings may be attributed to either the relatively low inclusion level of HWS in the experimental diets or the insufficient adaptation period, both of which might have been inadequate to exceed the response threshold required to alter the core rumen microbiota in ruminants. An important finding of this study was that the beta diversity of the ruminal microbiota, assessed by PCoA, did not exhibit significant separation among dietary groups. This indicates that the overall architecture of the microbial community remained largely resilient to the graded replacement of conventional feed with HWS. This phenomenon could be attributed to the fact that HWS primarily provides highly fermentable nutrients that may stimulate the growth of certain pre-existing, opportunistic taxa within the rumen, without drastically altering the fundamental interactions between broader microbial groups. Therefore, the ecological implications of HWS should be interpreted as a modulation of community membership within a stable structural framework, highlighting the need to focus on specific functional taxa rather than overall community topology when evaluating its impact. The dominant bacterial phyla in the rumen were Bacteroidetes (34.5%) and Firmicutes (55.1%), aligning with previous ruminant studies (Qian et al., 2018). Substitution of 5–20% HWS in the basal diet increased the relative abundance of Firmicutes while reducing Bacteroidetes and *Prevotella*. *Prevotella* is the most abundant genus within *Bacteroidetes* and plays a critical role in protein and carbohydrate degradation, particularly in polysaccharide and proteolytic processes (Kim et al., 2011). Elevated cellulose levels from HWS supplementation mitigated the decline in the relative abundance of *Prevotella* and stabilized ruminal pH. As expected, the cellulolytic bacteria, including *Ruminococcus, Succinivibrio*, and *Veillonella*, were enriched by increasing HWS content in the feed. *Ruminococcus* primarily degrades cellulose, while *Succinivibrio* produces succinate as a precursor for propionate and acetate synthesis. Propionate serves as a gluconeogenic substrate, absorbed by rumen epithelial cells as lactate and metabolized in the liver via gluconeogenesis or the tricarboxylic acid cycle. Propionate production via succinate fermentation and pyruvate utilization by *Veillonella* were correlated with increased propionate concentrations in this study (Van Gylswyk, 1995). Increasing HWS inclusion enhanced the relative abundance of energy-utilizing taxa within Firmicutes, promoting the proliferation of *Succinivibrio* and *Veillonella*. The high cellulose content in HWS stimulated robust cellulolytic activity, with fiber degradation by hemicellulases and cellulases, followed by further fermentation to volatile fatty acids, driving elevated propionate production (Abdel-Wareth et al., 2023).

Carcass performance serves as a critical metric for evaluating meat-producing livestock output value, primarily assessed through parameters including carcass weight (CW), dressing percentage, loin eye muscle area, and GR value. CW reflects meat yield and carcass quality, while loin eye muscle area strongly correlates with muscularity, and GR value indicates intramuscular fat deposition. (Sultana et al. 2022) reported that 25–75 g/kg hydroponic wheat seedling supplementation in broiler diets increased pre-slaughter weight, CW, breast muscle yield, and wing weight without affecting dressing percentage. Similarly, (Devendar et al. 2020) observed enhanced pre-slaughter weight and HCW in goats given feed containing 20% hydroponic barley seedlings substitution, though dressing percentage remained unchanged. In our study, 5–20% HWS substitution in a TMR diet elevated pre-slaughter weight and CW in Hu sheep, aligning with these findings. It is worth noting that the 15% and 20% HWS groups exhibited increased loin eye muscle area, potentially attributable to the high digestibility of HWS and the resulting optimal rumen fermentation. This improvement in the rumen microenvironment enhances production of propionate and other volatile fatty acids, thereby providing valuable energy substrates for muscle growth and reducing protein catabolism (Bergman, 1990).

Water-holding capacity (WHC), defined as muscle tissue's ability to retain intrinsic moisture under mechanical, thermal, or storage stress, is routinely assessed through drip loss and cooked meat percentage—critical parameters for evaluating meat processing suitability and shelf-life (Warner, 2017). In this study, 10% and 15% HWS substitution significantly reduced *longissimus dorsi* water loss rate, while all HWS groups exhibited elevated cooked meat percentage. These effects may stem from HWS-enhanced protein digestibility, which promotes nitrogen retention, increased myofibrillar protein content, and enhanced water-binding capacity via collagen-myoprotein interactions. The heat-induced protein denaturation during cooking can also form a hydrated matrix that preserves moisture, enhancing juiciness and flavor, key determinants of consumer preference in meat quality (Ebeid et al., 2022). This phenomenon may also be attributed to alterations in dietary starch composition resulting from HWS substitution, which could influence cellular energy supply pathways and consequently affect cellular water transport capacity (Boukrouh et al., 2024). The variation in cooked meat percentage may be associated with flavonoids in HWS, which influence the permeability of muscle cell membranes, allowing calcium ions to infiltrate the cells, thereby reducing glycolytic efficiency and making muscle proteins more prone to denaturation during boiling, consequently affecting cooked meat percentage (Li et al., 2025). Inosine monophosphate (IMP), a nucleotide metabolite and key umami compound, contributes to meat flavor through Maillard reaction-derived volatile aromatics; the level of its degradation products (e.g., hypoxanthine) directly correlates with a greater sense of palatability (Belloir et al., 2025). The *longissimus dorsi* muscle in the 20% HWS group exhibited significantly reduced IMP content, which attenuated umami intensity (characterized by savory, broth-like flavor profiles). Concurrently, hypoxanthine accumulation may have contributed to increased bitter off-flavors, a phenomenon that could be associated with post-slaughter pH changes. We propose a hypothetical pathway based on the established literature: during rigor mortis progression (ATP → ADP → AMP → IMP → inosine), a potentially lower ultimate muscle pH in sheep fed 20% HWS might have created a weakly acidic environment that destabilizes IMP. This could theoretically accelerate its enzymatic hydrolysis by phosphomonoesterase into inosine and subsequent breakdown to hypoxanthine and ribose via nucleoside hydrolases—a catabolic cascade known to be pH-dependent, which would limit IMP retention (Kanokruangrong et al., 2025). If this proposed pathway holds true, the degradation of IMP would attenuate umami intensity (typically manifested as savory, broth-like flavors), while simultaneously increasing the potential for an undesirable bitter taste due to hypoxanthine accumulation. However, this specific mechanistic cascade remains to be directly validated in future studies.

Lamb meat is valued as a high-protein, low-fat animal product with a distinctive flavor, largely deriving its nutritional and sensory qualities from its composition of amino acids and fatty acids (Ma et al., 2020). While 5–20% HWS substitution in the basal diet did not significantly alter amino acid profiles in *longissimus dorsi*, the numerical increases in Gly, Ser, Thr, Ile, Asp, Met, Tyr, and SEAA suggest sub-threshold modulation of amino acid metabolism. This is likely due to the highly conserved nature of muscle protein structure, which is genetically determined. In ruminants, the extensive modification of dietary protein by rumen microbes results in a relatively consistent post-ruminal supply of amino acids, unless the diet induces a major microbial shift. The improvements from HWS supplementation therefore appear to have been driven by enhanced nutrient availability and ruminal efficiency, which supported greater overall protein deposition without altering the fundamental amino acid profile of the deposited tissue. UFAs, prone to oxidative degradation, generate volatile aldehydes, ketones, and alcohols that contribute to lamb's characteristic ‘gamey' flavor, whereas SFAs with higher melting points enhance succulence through thermal stabilization during cooking (Saengsuk et al., 2024). Heptadecanoic acid (C17:0), an odd-chain SFA serving as both a meat quality biomarker and flavor modulator in ruminant adipose tissues (Munezero et al., 2024), was significantly elevated in HWS-fed lambs. This increase not only enhanced meat flavor characteristics but also improved lipid metabolism. Furthermore, long-term consumption of C17:0-enriched products may confer cardiovascular disease risk reduction benefits to consumers. This C17:0 accumulation may stem from bioactive, HWS-derived amino acids that affect propionate metabolism. As an odd-chain fatty acid, C17:0 deposition is tightly regulated by ruminal propionate flux—a process potentiated in T15 sheep by markedly elevated NH_3_-N concentrations, indicative of enhanced microbial deamination. Proliferation of ammoniagenic bacteria such as Clostridium glutamicum, likely accelerated α-ketoglutarate release into the tricarboxylic acid (TCA) cycle, stimulating propionate synthesis (Halarnkar and Blomquist, 1989). However, there is a lack of direct experimental evidence for transcriptomic or metabolomic analysis. This may provide a new idea for the future verification of key metabolic pathways in rumen and host tissues through multi-omics technology. Hepatic gluconeogenic conversion of propionate to glucose, coupled with its role as a methylmalonyl-CoA precursor for odd-chain fatty acid elongation, correlates with T0′s C17:0 peak. However, HWS substitution >10% linearly increased acetate production, altering the acetate/propionate ratio to suppress propionate-dependent Prevotellaceae_UCG_001 activity while activating acetate-generating fibrolytic taxa, like Ruminococcus flavefaciens, thereby reducing C17:0 synthesis (Zhang et al., 2017). The quadratic response of PUFA content to HWS inclusion may reflect polyphenol-mediated rumen microbial modulation. Ferulic acid and vitamin E in HWS likely protected cis-double bonds in α-linolenic acid (C18:3n-3) from biohydrogenation by inhibiting hydrolases of *Butyrivibrio* proteoclasticus (Banakar et al., 2023). Concurrently, Bacteroidetes-enhanced polysaccharide degradation increases short-chain fatty acid (SCFA) production, upregulating fatty acid-binding protein 2 expression to improve PUFA absorption (Li et al., 2023). Fiber-derived metabolites from the *Rikenellaceae*_*RC*9_*gut*_*group* may further activate peroxisome proliferator-activated receptor alpha, promoting PUFA transport into muscle (Mao et al., 2025). With 15–20% HWS, prolonged digesta retention could activate Desulfobacterota-mediated sulfate reduction, and generate hydrogen sulfide that would inhibit mitochondrial β-oxidation and PUFA translocation (Liu et al., 2024). Simultaneously, Firmicutes-enriched microbiota likely enhanced stearoyl-CoA desaturase activity, converting C18:3n-3 to stearic acid (C18:0), a process compounded by Proteobacteria-associated biohydrogenation, collectively driving the quadratic decline in PUFA deposition.

## Conclusions

This study demonstrates that a 15% replacement of conventional feed with hydroponic wheat seedlings in Hu sheep diets optimizes slaughter performance and economic returns, while higher substitution rates (20%) negatively impact these parameters and impair inosine monophosphate synthesis in meat. Rumen fermentation profiles and slaughter indices indicate hydroponic wheat seedlings supplementation enhances nitrogen utilization efficiency and growth performance in finishing Hu sheep, with the increased *Prevotellaceae*_*UCG*-001 abundance suggesting improved heptadecanoic acid synthesis in *longissimus dorsi* muscle through rumen microbial metabolic functions. The elevated pre-slaughter live weights and reduced *Butyrivibrio* and *Saccharofermentans* abundances further support enhanced feed efficiency. We recommend 15% hydroponic wheat seedlings inclusion as an optimal feeding strategy for finishing Hu sheep, offering potential benefits including increased production yield, reduced feeding costs, and alleviated human-livestock feed competition. This approach shows particular promise for implementation in cold regions, seasonally water-scarce areas, and intensive commercial farming systems to reduce land and water resource dependence.

## Data Availability

The datasets presented in this study are publicly available. This data can be found here: https://www.ncbi.nlm.nih.gov/sra, Accession Number PRJNA1262813.
